# Degradation of Xenobiotic Pollutants: An Environmentally Sustainable Approach

**DOI:** 10.3390/metabo12090818

**Published:** 2022-08-31

**Authors:** Rashi Miglani, Nagma Parveen, Ankit Kumar, Mohd. Arif Ansari, Soumya Khanna, Gaurav Rawat, Amrita Kumari Panda, Satpal Singh Bisht, Jyoti Upadhyay, Mohd Nazam Ansari

**Affiliations:** 1 Department of Zoology, D.S.B Campus, Kumaun University, Nainital 263002, Uttarakhand, India; 2 Department of Pharmaceutical Sciences, Sir J. C Bose Technical Campus, Bhimtal, Nainital 263136, Uttarakhand, India; 3 Department of Forestry and Environmental Science, D.S.B Campus, Kumaun University, Nainital 263002, Uttarakhand, India; 4 Department of Anatomy, Banaras Hindu University, Varanasi 221005, Uttar Pradesh, India; 5 Department of Biotechnology, Sant Gahira Guru University, Ambikapur 497001, Chhattisgarh, India; 6 Department of Pharmaceutical Sciences, School of Health Sciences and Technology, University of Petroleum and Energy Studies, Energy Acre Campus Bidholi, Dehradun 248007, Uttarakhand, India; 7 Department of Pharmacology and Toxicology, College of Pharmacy, Prince Sattam Bin Abdulaziz University, Al-Kharj 11942, Saudi Arabia

**Keywords:** xenobiotics, enzymes, microorganisms, metagenomics, sustainability

## Abstract

The ability of microorganisms to detoxify xenobiotic compounds allows them to thrive in a toxic environment using carbon, phosphorus, sulfur, and nitrogen from the available sources. Biotransformation is the most effective and useful metabolic process to degrade xenobiotic compounds. Microorganisms have an exceptional ability due to particular genes, enzymes, and degradative mechanisms. Microorganisms such as bacteria and fungi have unique properties that enable them to partially or completely metabolize the xenobiotic substances in various ecosystems.There are many cutting-edge approaches available to understand the molecular mechanism of degradative processes and pathways to decontaminate or change the core structure of xenobiotics in nature. These methods examine microorganisms, their metabolic machinery, novel proteins, and catabolic genes. This article addresses recent advances and current trends to characterize the catabolic genes, enzymes and the techniques involved in combating the threat of xenobiotic compounds using an eco-friendly approach.

## 1. Introduction

In the industrial revolution and urbanization era, the global environment’s poisoning by a complex mixture of xenobiotics has become a major environmental threat worldwide [1,2]. Xenobiotic contaminants such as azodyes, phenolics, polycyclic aromatic hydrocarbons (PAHs), halogenated compounds, personal care products (PCPs), pharmaceuticals’ active compounds (PhACs), pesticides, nitroaromatic compounds, triazines, and chlorinated compounds adversely affect the environment by their long-term persistence and slow or no biodegradation in the ecosystems [3,4,5]. Once xenobiotics are discharged into the environment, they enter the food chain, causing harmful impacts at each trophic level and adversely affecting human and animal health. In 1960s, the discovery of DDT (dichloro-diphenyl-trichloroethane), and methyl mercury residues in fish and wildlife sparked public interest in the bioaccumulation of xenobiotic chemicals [6,7,8,9,10]. In addition, these pollutants have teratogenic, carcinogenic, mutagenic, and toxic effects on all organisms. Therefore, removing toxic undegradable xenobiotics from the environment is necessary [11,12]. These xenobiotic compounds have been degraded by physical and chemical methods such as coagulation, filtration, adsorption, chemical precipitation, electrolysis, and ozonation. However, it is not always cost-effective; lack of space, complicated procedures, stringent regulatory requirements imposed on decontamination by various countries, public dissatisfaction, waste disposal issues, and toxic by-products turn more hazardous than the parent compounds [2,13,14].

Over the past few decades, microbial-assisted degradation (bioremediation) of xenobiotic pollutants has evolved into the most effective, environment-friendly, cost-effective method for removing these noxious contaminants. Bioremediation is a method that involves the destruction, eradication, immobilization, or detoxification of a wide range of chemical waste and other harmful chemicals from the environment by an inclusive action of microorganisms. Bioremediation-related technologies include phytoremediation, rhizofilteration, bioaugmentation, biostimulation, landfarming, bioreactors, and composting. It is now gaining popularity; this method takes advantage of microorganisms’ metabolic capabilities to eliminate contaminants, making them the most appropriate and promising. Persistent organic pollutants (POPs) cleanup with microbial enzymes is eco-friendly, cost-effective, and inventive [15,16].

Various laws and rules have been formulated to address the problems of xenobiotics, and many patents have been adopted and are in use in the EU and around the world, with an increased focus on reducing xenobiotics from the environment in a way that is economically, environmentally, and socially acceptable and viable with reduced accumulation or generating other toxic components in nature [17]. Furthermore, patents are an accurate indicator of inventive activity and their implementation in the analysis of xenobiotics and other harmful products could help scientists, stakeholders (technologists, business leaders, attorneys), policymakers, and researchers to gain access to technology updates, develop new processes and products, design future research strategies, and make critical decisions for developing R&D investment plans for more significant economic and environmental growth [18]. This review aims to convey up-to-date knowledge on recently identified catabolic genes for xenobiotic pollutants using various omics technologies. In addition, this review gives a concise note on the role of microbial enzymes in the detoxification of xenobiotics and also highlights various patents filed for the transformation of xenobiotics from various environments.

## 2. Xenobiotic Pollution and Its Impact on the Environment

Xenobiotic pollution of the environment is a global concern caused by anthropogenic activities such as urbanization and population expansion. The enormous amounts of harmful compounds released into the environment result in widespread ecosystem contamination. Prominent substances such as polycyclic aromatic hydrocarbons (PAHs), heavy metal ions, pesticides, fertilizers, and oil derivatives are found in soil, sediment, and water [4].

During the Industrial Revolution, scientific and technological advances became a source of people’s over-exploitation of resources, which destroyed various ecosystems [19]. The irrational use of human, veterinary drugs and pharmaceutical waste is another well-known contributor to environmental contamination. Compared to other chemical compounds, medicines potentially impact aquatic flora and fauna. However, pharmaceuticals are believed to cause only a minimal risk of acute environmental toxicity. The scenario may differ for chronic effects; nevertheless, there is a substantial dearth of evidence about chronic effects and their toxicity. Furthermore, there is little or no evidence of multi-generational life cycle consequences, even though many aquatic creatures are exposed to toxicity throughout their life [20].

**Figure 1:**
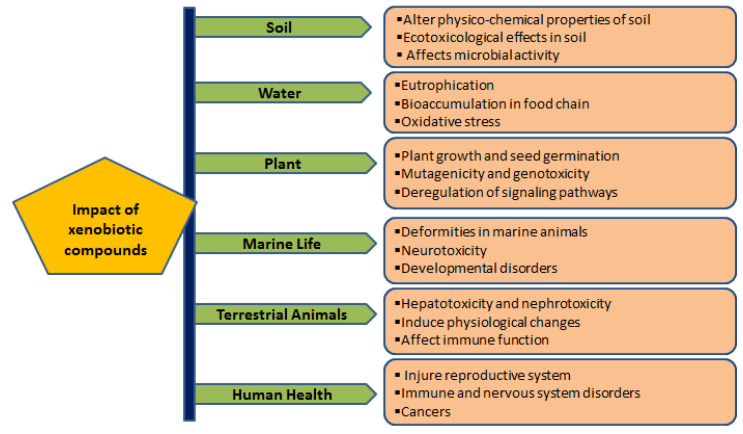
Hazardous effects are caused by direct or indirect exposure of xenobiotic compounds on the environment, plants, animals and human health. Xenobiotics impose ecotoxicological effects on soil organisms, reduce microbial activity, and change the soil’s physico-chemical properties. Releasing xenobiotic compounds to aquatic systems (fresh and marine water) causes eutrophication and severe threats to faunal diversity, including deformities and developmental disorders. In addition, continuous exposure to xenobiotics adversely affects the immune, reproductive and nervous systems and sometimes causes various cancers.

Major xenobiotic compounds have hazardous effects on the environment, plants, animals, and humans (Table 1; Figure 1).

### 2.1. Impact of Xenobiotics on Soil

Xenobiotics such as dioxins, 1,1,1-trichloro-2,2-bis (4-chlorophenyl) ethane (DDT), polychlorinated biphenyls (PCBs), chlordane, polycyclic aromatic hydrocarbons (PAHs), and nitroaromatics are the primary threat to the soil ecosystems of developed nations. However, there are reports that a few other pollutants such as benzene, nitrobenzene, toluene, xylene, aniline, ethylbenzene, trinitrotoluene/dibenzofurans, and chlorinated solvents could be xenobiotic, especially in the soil ecosystem [32]. Cosmetics and personal care products also contribute as xenobiotic pollutants, especially parabens in soil and air [33] and azodyes in soil, due to one or more aromatic rings and azo bonds [34].

Anthropogenic activities that stimulate these chemical compounds in soil include industrial activities, fuel combustion, military movement, use of pesticides, fertilizers, and soil modifications in high-production agricultural practices that cause detrimental effects [35,36]. Chemical characteristics of xenobiotics and site conditions influence their bioavailability, and distribution in soil, with soil organic matter(SOM) playing an important role [37]. Pesticides (herbicides, insecticides, fungicides, algaecides, bactericides, etc.) are chemicals used for crop protection and management and are the most widely used toxins in the environment over the last century. Millions of tonnes of pesticides are produced and spread each year around the world [38]. Environmental factors such as temperature, soil pH, and moisture significantly impact the behavior of persistent organic pollutants (POPs) in the soil. One possible strategy is binding xenobiotic compounds to soil organic matter (SOM). Many xenobiotics and their degraded products resemble humic precursors and are frequently used in humification. It has been suggested that this naturally existing process is used to neutralize environmental contaminants found in soil. Inorganic minerals interact well with xenobiotics and play a crucial role in xenobiotic transformation [39].

### 2.2. Impact of Xenobiotics on Water

The diffusive and point contributions of anthropogenic activities such as urban industrial production, transportation, building construction, and housing pollute surface and groundwater in urban areas. The presence of chemical substances and indicators of human activity in urban water systems has been the subject of numerous kinds of research [40]. In sewage treatment plants, some common xenobiotics sensors must be treated with municipal wastewater before being discharged into aquatic systems. Several trace metals, xenobiotic substances, and synthetic organic chemicals, such as PAHs, phthalates, and pesticides, are also noticed in different water bodies [41]. Xenobiotic substances can enter water bodies through different sources. These include (a) airborne particulate deposition; (b) surface water running from roads and land surfaces; (c) continuous inputs from commercial and sewage effluents, as well as fossil fuel products; (d) solid waste burning [42]. Xenobiotics substances also reach the water table through the leaching process, which affects the biological integrity of aquatic ecosystems [20]. The presence of xenobiotic pollutants induces oxidative stress among aquatic organisms. A recent study by Ibor et al. [43] observed a significant increase in oxidative stress response in the fish fauna of an artificial Eleyele lake, Nigeria.

A study reported that xenobiotic compounds alter the homeostasis in fishes and cause oxidative stress by producing large numbers of reactive oxygen species and suppressing the antioxidant system [44]. 

### 2.3. Impact of Xenobiotics on Plants

Xenobiotics affect the plant’s physiological and morphological characteristics in many ways; for example, particulate matter from the automobile sector changes the photosynthetic pigments, protein, cysteine contents, leaf area, and the foliar surface of plants [45]. The extensive range of xenobiotics with diverse structures and designs causes changes in gene expression, regulation, and signal transduction in the higher plants. Xenobiotics, such as phytohormone analogs, have intrinsic interactions with plant hormone receptors and signaling pathways [46]. Metals that are needed for plant growth, such as Cu, Zn, Fe and Mo, have deleterious effects at high concentrations, but metals that are not essential for plant growth, such as Pb, Cd, Hg and As, have adverse effects even at low concentrations in plant growth [47]. Xenobiotics induce DNA damage in the case of plants due to the production of reactive oxygen species and oxidative stress. The signaling pathways get deregulated due to xenobiotic toxicity in plants by influencing various signaling receptors such as G-Protein coupled receptor and receptor tyrosine kinase [48].

### 2.4. Impact of Xenobiotics on Marine Life

Xenobiotics negatively impact several metabolic processes of marine animals, particularly in developing fish embryos, causing morphological and functional abnormalities, retarded growth leading to death. Altered body shape, body abnormalities, hatching delays, and death have also been recorded in fishes [49]. Dyes and paints are also considered xenobiotics because they restrict sunlight penetration and inhibit gas exchange even if they are present in the traces [50]. Pesticides and herbicides are significant sources of xenobiotic pollution in marine life. Chemicals, including organophosphorus, nitrophenols, morpholine, synthetic pyrethroids, and carbamates, are often used in agricultural and daily life; later on, these substances enter various water bodies, including the sea and ocean. Insecticide such as β-Cypermethrin is a severe threat to the life of marine life and invertebrates [51].

### 2.5. Impact of Xenobiotics on Terrestrial Animals

Xenobiotic exposure is also possible due to application or inoculation of pharmacologic drugs or other chemicals as part of a typical conditioning or experimental operation. The consumption pattern and disposition of xenobiotics determine their toxicity. In addition, the mechanical and chemical properties also play a vital role in determining the toxicity of these xenobiotics’ compounds [52]. The xenobiotics and their metabolites may induce physiological changes in animals by altering immunological functions, cardiovascular indices, or organ systems. For example, ivermectin, a popular anthelmintic and acaricide, is harmful to some dog breeds and mouse strains due to a lack of p-glycoprotein [53]. Compared to controls, pazufloxacin and meloxicam cause oxidative damage in rabbits, including decreased glutathione content and considerable lipid peroxidation [54].

### 2.6. Impact of Xenobiotics on Human Health

Xenobiotics pollute the environment, so their assimilation by living species has increased dramatically in recent decades. Introducing these substances into ecosystems may increase allergic reactions, organism mortality, genetic alterations, immune system lowering, metabolic disorders, and disruptions in natural ecosystem processes [55]. Humans are exposed to a wide range of xenobiotics, such as medications and non-essential exogenous substances, throughout their lives by ingesting, breathing, dermal contact, or any other intravenous route of exposure that may represent a risk to human health [56]. Xenobiotics may alter the human gut microbiome leading to dysbiosis, which is indirectly linked to various undesirable health outcomes. The continuing biotransformation process consistently seeks to balance the metabolic activation of xenobiotics to the detoxification of their mutagenic metabolites, as it evolved to neutralize and remove body-invading agents. When this balance is disrupted, chronic diseases and DNA damage in the human body can occur. The toxicity of xenobiotics varies significantly between individuals. These oscillations are caused by the organism’s enhanced sensitivity and intraspecific variability. A large spectrum of substances is utterly foreign to the human body. These chemicals have harmful and irritating effects on various human organs and systems directly and indirectly [57].

## 3. Omics Approaches to Combat Xenobiotic Pollution

**Figure 2:**
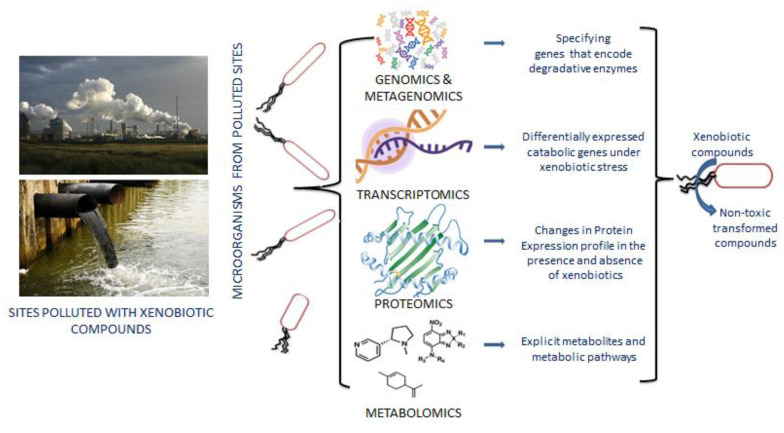
Distinct features of multi-omics technologies in the transformation of xenobiotic compounds. Genomics and metagenomics identify detoxifying enzymes from the whole genome or metagenome sequencing data. RNA seq or transcriptomics data indicate up- and down-regulated genes in response to xenobiotic exposure. Proteomics techniques help to compare the changes in protein profile in the presence and absence of toxic compounds.

Human activities regularly emit xenobiotics into the environment, causing pollution and harming human and natural ecosystems. However, certain xenobiotic-degrading bacteria and fungi have been identified. Most of the xenobiotic-degrading bacterial strains rely only on xenobiotics for their carbon source and energy, making them great models for studying bacterial adaptability and evolution in the environment (Figure 2) [58]. Initially, bacterial strains with metabolic properties were isolated and cultured to degrade pollutants. However, very few microbes are cultivable with xenobiotic degradative potential; few of them have been isolated and characterized in the recent past with incomparable biodegradation ability such as *Alcaligenes* [59], *Pseudomonas* [60], *Enterobacter*, *Achromobacter*, *Hyphomicrobiaceae*, *Microbacterium* [61], *Micrococcus and Rhodococcus* [62], *Aeromonas* [63], *Sphingobium* [64], *Aspergillus* and *Purpureocillium* [65], *Penicillium* and *Trichoderma* [66], *Rhodotorula* and *Candida* [67] etc. Hence, new culture-independent approaches such as metagenomics are gaining momentum to identify non-cultivable microbes with xenobiotic degradation potential [68,69]. Few relevant xenobiotic degrading microorganisms were identified with culture-independent approaches, such as *Sphingopyxis*, *Afipia*, *Oligotropha*, *Rhodopseudomonas*, *Mesorhizobium*, and *Stenotrophomonas* [70]. The dominance of *Thalassolituus* and *Oleispira* have also been identified as vital oil-degrading bacteria through metagenomics and the metatranscriptomic approach [71].

### 3.1. Genomics and Metagenomics

Genome sequencing of uncultured microorganisms helps to find new genes associated with the microbe and gives details of the degradation potential of these microbial communities. Genomics determines the genetic information and metagenomics determines the genetic sequences of a community of an organism in total. Internal transcribed spacer (ITS) regions distinguish fungal DNA from other organisms in the ribosomal genes. Plants or bacteria do not share these regions. Thus, ITS amplicon sequencing helps identify fungal species able to degrade xenobiotic compounds [72]. Functional metagenomics studies demonstrated that *Burkholderia, Bradyrhizobium, Koribacter* and *Acidomicrobium* were the most abundant genera in soil contaminated with pesticides [73]. This study also reported the abundance of phosphodiesterase encoding genes that plays a vital role in organophosphorus degradation. Whole-genome sequencing studies of atrazine-degrading *Pseudomonas* sp. Strain ADPe, *Variovorax* sp. Strain 38R, *Arthrobacter* sp. Strain TES, *Chelatobacter* sp. Strain SR38 [74] using Illumina HiSeq 3000 platform unravel the genetic changes in the strains during environmental challenges.

The *Gordonia* sp. 1D genome analysis revealed the existence of two alkane hydroxylase gene clusters, dibenzothiophene cleavage genes, and intermediates in the metabolism of salicylate and gentisate-naphthalene. In hot climates, the highly effective thermotolerant strain *Gordonia* sp. 1D can be employed to remediate oil-contaminated soils [75]. Complete genome sequence data for several significant microbial strains, including Shewanella oneidensis MR-1, Pseudomonas aeruginosa KT2440, Deinococcus indicus R1, and Dehalococcoides mccartyi WBC-2, have already been provided, which is crucial for efficient bioremediation (http://www.tigr.org, accessed on 20 February 2022).

The metagenomic approach is called ecogenomics, community, or environmental genomics [68]. Metagenomic approaches can link microbial identity, functional diversity, and the role of essential genes, for which metagenomic libraries are constructed. Although sequence-driven and function-driven approaches are used for diversity screening, novel gene identification and functions are being studied in a new approach called function-driven metagenomics. Low recovery of active clones is the main limitation of this approach [76,77,78].

### 3.2. Transcriptomics and Metatranscriptomics

A subset of genes transcribed to RNA is referred as transcriptome and links the genome, the proteome, and the cellular phenotypes. The mRNA expression level, which is upregulated or downregulated in an organism, can be determined using RNA sequencing and DNA microarrays [79,80]. The mRNA expression level changes with the environmental conditions which the organisms inhabit; the high cost, tremendous efforts, and a smaller number of genes to be analyzed limits the use of DNA microarray [79]. Also, when interpreting the microarray data statistically, there are chances of false results [81]. RNA sequencing has the edge on DNA microarrays due to a more comprehensive quantitative range of expression [82]. Hence, many studies are now relying upon this particular approach. The transcriptomic study of a DDT-resistant *Trichoderma hamatum* FBL 587 showed upregulation of around 1706 genes involved in DDT degradation and upregulation of many DDT-metabolizing enzymes such as FAD-dependent monooxygenases, epoxide hydrolases, glycosyl- and glutathione-transferases [83]. Lima-Morales et al. [84] investigated the catabolic gene diversity of BTEX-contaminated soil under continuous long-term pollutant stress to identify the occurrence of important genes for catabolic pathways. The RNA-seq and coexpression network analysis approach was used to reveal the metabolism of hexabromocyclododecane degradation in *Rhodopseudomonas palustris* [85]. Lima-Morales et al. further confirmed the over-expression of hexabromocyclododecane degradation enzymes such as glutathione-S-transferase, haloacid dehalogenases, cytochrome p450, dioxygenases and transcriptional regulator LysR by qRT-PCR. The mechanism of breakdown of organophosphorous pesticide phoxim by *Bacillus amyloliquefaciens* YP6 and its biodegradation pathway was proposed based on the transcriptomic data [86]. They observed the upregulation of oxidase, hydrolase and NADPH- cytochrome P450 reductase genes for hydrolysis, oxidation and dealkylation of phoxim. Metatranscriptomic analysis of a two-cell Canadian biobed system identified diverse xenobiotic-degrading bacterial phyla such as *Sphingopyxis*, *Mesorhizobium*, *Oligotropha*, *Stenotrophomonas*, *Afipia and Pseudomonas* having an important role in the degradation of xenobiotics [70].

### 3.3. Proteomics and Metaproteomics

Proteomics is the study of all the proteins expressed in an organism, and metaproteomics/community proteomics is the large-scale study of identifying and quantifying proteins from microbial communities [87]. Protein synthesis, protein-protein interaction, mRNA turnover, and gene expression-related studies can be performed using Proteomics.

A comparative proteomic analysis study of the strain *Burkholderia zhejiangensis* CEIB, S4–3 in the absence and presence of methyl parathion, revealed the changes in protein expression profile through 2D-PAGE [88]. The MALDI-TOF approach was used to identify 72 differentially expressed proteins; 35 and 37 in the absence and presence of methyl parathion, respectively. They also concluded that these proteins are involved in catabolism of aromatic compounds and detoxification of xenobiotics. The metaproteomic approach used by An et al. [89] indicated the upregulation of 430 proteins which are mainly involved in the detoxification of Direct Black G azo dye, such as peroxidase, aldehyde dehydrogenase and oxidoreductase activity proteins.

### 3.4. Metabolomics

This approach involves the analyses of primary and secondary proteinaceous metabolites produced by microbial cells under defined physiological conditions. Metabolites produced by microbes play an essential role in intra-species and inter-species interactions. Various methods can study metabolomics, such as metabolic flux analysis, metabolite profiling, metabolic fingerprinting, and target analysis, to identify and quantify a wide array of cellular metabolites [90].

Metabolomics, or global profiling of metabolite content, is a potent tool used to investigate toxicant effects on organisms. The metabolic approach involves analyzing primary and secondary proteinaceous metabolites inside the cells, tissues, or bio-fluids. Metabolomics is the study of metabolites in biological matrices under specified conditions. Metabolomics has recently been utilized in environmental studies to investigate metabolic alterations in humans and other creatures exposed to various contaminants. Thus, metabolomics has become an essential technique in research to investigate xenobiotics’ molecular effects [91].

In the metabolism of any xenobiotic compound, a series of metabolic pathways utilizing a variety of enzymes is needed [92]. Recent genome analyses of bacterial strains that digest xenobiotics have suggested that they arose recently by gathering genes for xenobiotic degradation, with mobile genetic components playing a pivotal role in gene recruitment [93]. However, the origins of such bacterial strains’ genes and evolutionary processes are mainly unclear. The xenobiotic degrading enzymes are valuable for studying protein evolution since they have a wide range of activities and their characteristics vary substantially with a limited number of mutations [94].

The metabolomics approach was used to study the degradation mechanism of carbaryl and other N-methyl carbamates pesticides in *Burkholderia* sp. strain C3 and the findings of this study demonstrated *Burkholderia* sp. C3’s metabolic adaptation to carbaryl in comparison to glucose and nutrient broth. The metabolic changes were most prominently linked to the biosynthesis and metabolism of amino acids, sugars, PAH lipids and cofactors [95]. In addition, a comparative metabolic approach was used to examine the microbial breakdown of cyfluthrin by *Photobacterium ganghwense* [96]. Soil metabolomics is an efficient method for elucidating the intricate molecular networks and metabolic pathways utilized by the soil microbial community. This method can also be used to identify soil pollution biomarkers [97].

The metabolomic characterization of two potent marine bacterial isolates, *Mycobacterium* sp. DBP42 and *Halomonas* sp. ATBC 28, is capable of degrading phthalate and plasticizers such ATBC, DBP and DEHP. They concluded that DBP is degraded by sequential elimination of the ester side chains and produces monobutyl phthalate first then phthalate and two butanol molecules by employing a metabolomics approach [98]. *Drechslera* sp. strain 678, is capable of degrading a common additive used in gasoline, known as methyl tertiary-butyl ether (MtBE), the organic extracts obtained from the culture filtrate of strain 678 were examined. The presence of two major bioactive metabolites, monocerin and an alkyl substituted epoxycyclohexanone derivative with good antifungal activity and bioremediation, was revealed by metabolomic analysis [99].

Metabolomics and bioinformatics technologies and databases have improved the knowledge of microbial communities, their catabolic pathways, and the genes encoding catabolic enzymes. Thus, it is an effective method for identifying novel metabolic pathways and describing metabolic networks. It has been used to evaluate variation in metabolic and catabolic gene expressions, analyze the physiology of microbial communities in varied environments, and uncover the bacterial species for xenobiotic pollutant destruction. The advance in various omics technologies such as whole genome sequencing, shotgun metagenome sequencing, transcriptomics analysis and metabolomics identified many xenobiotic-degrading microorganisms and their catabolic genes (Table 2). A recent study on the transcriptomics of *Fusarium verticillioides* identified genes (FDB1 and FDB2) and four associated putative gene clusters involved in the degradation of lactam and lactone xenobiotics. The study also reported the induction of a gene cluster involved in the biosynthesis of vitamin B6 upon exposure to 2-benzoxazolinone and it helps the fungus to combat the ROS generated during the metabolization of xenobiotic compounds [100]. The omics approaches clarify our understanding that many putative gene clusters are induced not only to catabolize the xenobiotics directly but also that their expressions are related to many intermediates generated during the degradation pathways.

#### 3.4.1. Analytical Approaches for Metabolite Screening and Their Use in the Detection and Degradation of Xenobiotics

**Figure 3:**
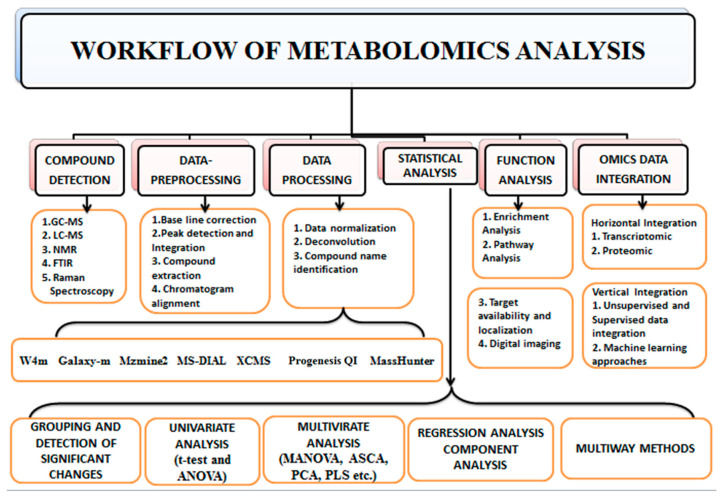
Workflow of Metabolomics. The first step of the metabolomics workflow is compound detection; by employing mass spectrometry, NMR, FTIR, etc. The second step is data pre-processing, which aims to improve the signal-to-noise ratio and quality of spectra by noise reduction, baseline correction, peak detection and integration. The third step is data processing through data normalization to reduce technical bias through various software such as MZmine, XCMS, Progenesis QI, etc. The fourth step is a statistical analysis to detect the expressed metabolite, followed by the fifth step, which is function analysis that interconnects metabolites to biological pathways. The final step is integrating metabolomics data to omics data (omics data integration) to understand the mechanism of action.

The characteristics of metabolomics data require the implementation of several tools of bioinformatics by a particular workflow. Various approaches are utilized to separate and characterize distinct metabolite classes (Figure 3). The major analytical techniques of metabolomic investigations are high-throughput techniques such as GC (Gas chromatography), HPLC (High-performance liquid chromatography), UPLC (Ultra-performance liquid chromatography), and CE (Capillary electrophoresis) with MS (mass spectroscopy) and NMR spectroscopy which enable the isolation, detection, characterization, and quantification of such metabolites and associated metabolic pathways [51,106]. Plumb et al. [107] first combined the multivariate data analysis and LC-MS to detect xenobiotics metabolites; numerous xenobiotic investigations have used UPLCMS-based metabolomics for further studies. Among different analytical techniques, LC-MS (Liquid chromatography-mass spectroscopy) and NMR have been employed extensively in metabolomic studies [108,109,110]. Many analytical procedures are generally required to achieve comprehensive data due to the metabolites’ diverse chemical characteristics. A single extract of metabolites from biological materials can contain thousands of metabolites. In untargeted metabolomics, it is typically required to segregate metabolites using an analytical column based on their chemical characteristics [106].

Many researchers have found these techniques very helpful in identifying substances and metabolites useful in the detection and degradation of xenobiotics, ref. [111] identified three oxidative products and two cellular metabolites by Gas Chromatography-Mass Spectrometry capable of debromination and mineralizing 2, 4, 6-tribromophenol (TBP). Chen and Kim [108] used LC-MS, for metabolomic investigations of XIT (xenobiotic-induced toxicities). Rodríguez-Robledo et al. [112] determined endocrine disruptors atrazine and propazine metabolites in seminal human plasma by LC-ESI-MS/MS. Lee et al. [113] analyzed the proteome of the PAH-degrading bacterium *Sphingobium chungbukense*. This strain displayed exceptional aromatic compound destruction capabilities and it was also observed that 2-DE and MALDI-TOF-MS effectively analyze xenobiotic chemicals such as phenanthrene, naphthalene, and biphenyls (PNB), and their related proteins. The 5-carboxylated diclofenac could be a crucial intermediary for the complete biodegradation of diclofenac (xenobiotic) via 2,6- dichloroanailine and 3-(carboxymethyl)-4-hydroxybenzoic acid by a microbial consortium. The carboxylated diclofenac intermediate could be extracted and identified by LC-MS/MS-TOF [114]. Bhattacharyya et al. [115] implemented modified QuEChERS-GC-MS-LC-MS/MS technique for screening several classes of multiple pesticides in betelvine and estimating public risk.

#### 3.4.2. Miscellaneous Methods Used in Detection of Xenobiotics

Appropriate extraction and analytical methods for the separation and determination of xenobiotic and derivative mixtures are critical, and they must be fast, accurate, and affordable [17]. In the recent past, there has been noticeable progress in the development of sample preparation techniques such as quick, easy, cheap, effective, rugged, and safe (QuEChERS), dispersive liquid-liquid microextraction (DLLME), focused ultrasonic solid-liquid extraction (FUSLE), solid phase extraction (SPE), solid phase microextraction (SPME), stir bar sorptive extraction (SBSE), hollow-fiber liquid phase microextraction (HFLPME) and many others [116].

QuEChERS analyzes multi-residue pesticides, antibiotics, hormones, mycotoxins, polycyclic aromatic hydrocarbons, and persistent organic pollutants such as dioxins and polychlorinated biphenyls in food and environmental matrices. QuEChERS is paired with GC–MS or LC–MS for high selectivity, sensitivity, and specificity [117]. Solid phase extraction (SPE) encompasses preparation strategies for organic pollutants from environmental matrices. Pharmaceuticals, pesticides, carbamate, bisphenols, and phthalate acid esters are analyzed using this technique [118]. In contrast, solid-phase microextraction (SPME) allows simultaneous sampling and sample preparation and is used to analyze pesticides, polycyclic aromatic hydrocarbons, phenols, amines, and polychlorinated bisphenols in food and environmental samples [119]. The stir bar sorptive extraction (SBSE) is used to determine pesticides, pharmaceuticals, polycyclic aromatic hydrocarbons, phenols, alkylphenols, chlorophenols, bisphenol A, and mycotoxins present in the environment and food [120].

HFLPME with a porous hollow-fiber membrane is used to analyse lead, arsenic, medicines, and other organic substances in environmental, clinical, and biological samples, petroleum products, pharmaceuticals, and food. It works with chromatography, electrophoresis, molecular and atomic spectrometry, and electrochemistry instruments [121]. DLLME is applied for organic compounds such as phthalate esters or parabens and metal ions such as cadmium, selenium, and lead. Pesticide analysis is used to look for chlorophenols and endocrine-disrupting phenols and medicines [122]. FUSLE can identify inorganic, organometallic, and organic substances in environmental samples, such as polycyclic aromatic hydrocarbons, PCBs, phthalate esters, and nonylphenols. It can also detect endocrine disruptors (bisphenol A and alkylphenols) in sewage sludge [123].

## 4. Role of Microorganisms in Xenobiotic Degradation

**Figure 4:**
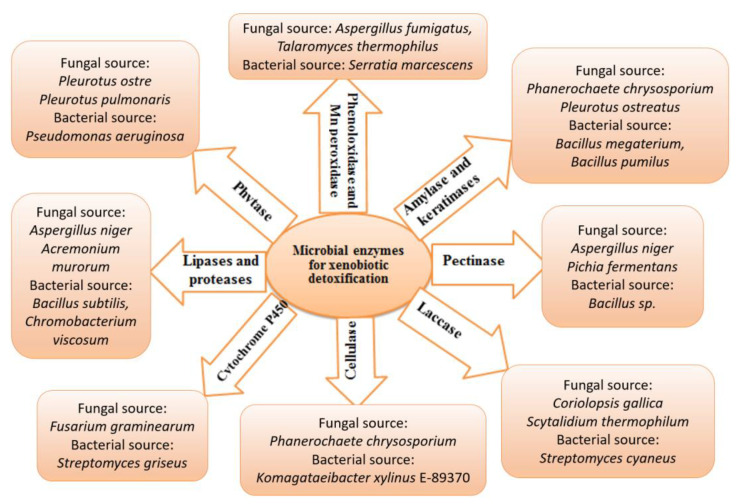
Microbial enzymes in xenobiotic management. This figure summarizes a few representative enzymes and their corresponding microbial sources involved in xenobiotic detoxification.

Chemical contamination can be cleaned up using biological organisms in a process known as bioremediation. The biotransformation of xenobiotics in soils, sediments, and water bodies relies heavily on microorganisms. Bioremediation uses the biological systems of living creatures (bacteria, fungi, and plants) and enzymes [124,125]. Microorganisms have an incredible ability to catabolize with the help of various genes, enzymes, and degradation pathways involved in biodegradation. Numerous microbes such as *Alcaligenes*, *Cellulosimicrobium*, *Microbacterium*, *Micrococcus*, *Methanospirillum*, *Aeromonas*, *Sphingobium*, *Flavobacterium*, *Rhodococcus*, *Aspergillus*, *Penicillium*, *Trichoderma*, *Streptomyces*, *Rhodotorula*, *Candida* and *Aureobasidium* have been isolated, characterized and have exhibited an excellent ability to biodegrade a variety of xenobiotic pollutants found in soil/water settings [79]. However, few representative microbial enzymes are involved in detoxifying xenobiotics, including cytochrome P450s, laccases, cellulase, phytase, proteases, and lipases shown in Figure 4. These enzymes can degrade aromatic hydrocarbons, dyes and halogenated compounds through various mechanisms.

### 4.1. Xenobiotic Degrading Enzymes Associated with Bacteria

Bacteria are known for their extraordinary capacity to multiply rapidly in large numbers and withstand harsh environmental conditions [126]. Recent genomic investigations of strains of bacteria that digest xenobiotics suggest that they evolved by accumulating genes for xenobiotic destruction. Bacterial species such as *Pseudomonas*, *Escherichia*, *Sphingobium*, *Pandoraea*, *Rhodococcus*, *Gordonia*, *Bacillus*, *Moraxella*, *Micrococcus (aerobic bacteria)*, *Pelatomaculum*, *Desulfotomaculum*, *Syntrophobacter*, *Syntrophus*, *Desulphovibrio*, *Methanospirillum*, *Methanosaeta (anaerobic bacteria)*, etc., have been isolated from soil and characterized for their biodegradation potential of xenobiotic compounds (DDT, lindane, PCBs, TNT and crystal violet) [127]. The human intestinal microbiota has a direct xenobiotic-metabolizing potential, but it can also affect the expression of host metabolizing genes and the activity of host enzymes [79]. Based on the examination of 16S rRNA and gyrB gene sequences, strain 1D of thermotolerant bacteria isolated from oil-contaminated soil at a refinery was identified as *Gordonia* sp. [72].

Aromatic compounds (xenobiotics) act as an electron-donating substrate in the lack of oxygen (anaerobic condition), and microbes grow by oxidizing these substances in the existence of an electron acceptor. Enzymatic biodegradation begins with selecting an enzyme for a bioremediation application; it must be capable of degrading the target pollutants into less-toxic products [127]. Many bacteria species can potentially change the hazardous xenobiotic substances into less or nontoxic substances with the help of specific enzymes present inside them.

The present review aims to report recent investigations on microbial degradation of aliphatic and aromatic hydrocarbons. The biodegradation of different types of hydrocarbons requires distinct enzymes’ due structural variation of these xenobiotic compounds at a molecular level [128]. The degradation of aliphatic hydrocarbons occurs either through monooxygenases which add single oxygen to the terminal methyl functional group or dioxygenase, which adds two oxygen atoms resulting in the peroxide formation converted to a fatty acid. The fatty acid molecule oxidizes to form TCA cycle intermediates that further metabolize to CO_2_ and H_2_O. The aromatic hydrocarbons are slowly degradable due to low solubility, production of toxic metabolites and metabolite repression [129]. At first, these compounds are converted to cis-dihydrodiols and cleaved by dioxygenase enzymes either through ortho- or meta-cleavage pathways. Then, the fission of aromatic rings occurs between the hydroxyl groups in ortho-cleavage and adjacent to hydroxyl groups in meta-cleavage pathways, finally leading to intermediates of central pathways. A few recently isolated bacteria and their associated enzymes responsible for aliphatic and aromatic hydrocarbons along with their mechanism of action are listed in Table 3.

### 4.2. Xenobiotic Degrading Enzymes Associated with Fungi

In addition to bacteria, fungi have a role in organic pollutant remediation. They have unique characteristics that make them ideal microorganisms for bioremediation procedures. They can reduce pollutant concentrations by physically adsorbing various contaminants via a thick cell wall composed of polymers such as chitin and cellulose. The fungal decomposition of xenobiotic compounds has highlighted the importance of the intracellular enzymatic system’s involvement in xenobiotic transformation (Table 4) [79]. These fungi benefit various activities, including biofuel degrading, environmental management, and industries such as food, paper, beverages, textiles, etc.

## 5. Practical Use of Microorganisms in Bioremediation of Xenobiotics

The patents are highly relevant to xenobiotic degradation; many such patents were retrieved from different databases on the basis of priority of filing and properties relevant in use to handle xenobiotics. Therefore, the search includes publicly available databases, i.e., Espacenet, DPMA, USPTO, JPO, EPO, PatFT, WIPO which cover databases produced by the Canadian Intellectual Property Office, German patents, German Patent and Trademark Office, European Patents and Chinese Patents etc (Table 5).

Regarding the environmental threats of xenobiotic compounds, there are many proven methods and products in the form of patents and process patents [99,146,147,148,149]. However, with the fast-growing technologies and human needs, many products are being designed globally, and many are not entirely degradable; therefore, scientists are working on those with long shelf-life and poor degradative nature.

**Table 5:** Various patents and their properties used in the field of Xenobiotics.

Patent	Patent No.	Country	Application	Novelties/Inventions	References
Microbial degradation of waste/sludge	0 274 856 A1	England; European Patent	Biotransformation and/or mineralisation of each determined constituent of the waste	This study revealed the use of the defined assorted culture of bacteria isolated through enrichment on major individual constituents of an effluent, followed by mixing the isolates to detoxify the complex non-degradable effluent.	[150]
Microbial removal of xenobiotic dyes	DD290004A5	Germany; German Patent	Microbial degradation of xenobiotic dyes from triphenylmethane compounds	This invention is unique in terms of its way of selecting and using oleophilic microorganisms that ensure the degradation of xenobiotic dyes, in particular, those of triphenylmethane compounds	[151]
Microbial detoxification of xenobiotics using yeast	US4968620A	Peoria, United States; United States Patent	Detoxification of a variety of xenobiotics, including insecticides, herbicides, mycotoxins, and plant toxins (allelochemicals)	This invention provides insight into symbiotic yeast i.e., cigarette beetle (*Lasioderma serricorne*) NRRLY-18546 that detoxify pesticides, herbicides, mycotoxins, and plant poisons (allelochemicals)	[152]
Two-phase partitioning bioreactor for the degradation of a xenobiotech (organic and aqueous)	CA2216327A1	Canada; Canadian Intellectual Property Office	Causing the microorganism to metabolize the xenobiotic in the aqueous phase	The novelty of the invention is the two-phase concentration of xenobiotic compounds using bioreactors	[153]
Bioremediation of Xenobiotics Including Methyl Tert-Butylether	US 6,194,197 B1	United States; United States Patent	Degradation of Methyl Tert-Butylether (MTBE)	The novelty of this patent suggests that the co-metabolism of MTBE by graphium and other microbial species having a non-specific P-450 cytochrome oxidase could be used for the remediation of MTBE contamination	[154]
Treatment of contaminated groundwater using immobilized cells	WO 01/32566 Al	United States; Australian Patent	Creating a “bio-trench” or “bio-curtain” to clean contaminated groundwater	A method of removing contaminated groundwater is provided which places a biological permeable barrier in the path of the groundwater flow to contact the contaminated groundwater with encapsulated microorganisms which act to decontaminate the contaminated groundwater	[155]
Environmental remediation of organic compounds	EP 0 822 253 B1	Tokyo-Japan; European Patent	Biodegrading of chlorinated organic compounds such as trichloroethylene (TCE) and dichloroethylene (DCE)	Processes for making harmful chemical substances harmless or less harmful by effecting a chemical change in the substances by biological methods, i.e., processes of utilizing enzymes or microorganisms as whole	[156]
Microbial decomposition of xenobiotics	DE10125365A1	Germany; German Patent	Degradation of the herbicide Isoproturon	Effective method for decomposing xenobiotics (X) using a physiologically compatible combination of at least one fungus (A) with mono-/di-oxygenase activity and at least one fungus (B) with glutathione-S-transferase (GST) activity. An independent claim is also included for a combination of decomposing (X) containing (A) and (B).	[157]
Anaerobic microbial degradation of phthalic acid esters	WO2006136173A2	Denmark; World Intellectual Property Organization International Bureau	Degradation of phthalic acid esters	A process for anaerobic microbial degradation of phthalic acid esters, comprising the step of adding to a bioreactor at least one bacterial strain, which as a pure isolate capable of anaerobic degradation of phthalic acid esters.	[158]
bioremediation of chlorinated organic compound using recombinant bacteria	US 7,989,194B2	Chile; United States Patent	Degradation or mineralization of pollutants such as polychlorobiphenyls (PCBs),	*Wautersia eutropha strain* JMS34, a recombinant bacterium that can completely degrade or mineralize pollutants such as polychlorobiphenyls (PCBs), bioremediation of PCB-contaminated environments that contain a bacterial inoculum of this recombinant strain.	[148]
Method for simultaneous biological removal of nitrogen compounds and xenobiotics of wastewaters	WO2013166611	Prilly, Switzerland; European Patent	Removal of nitrogen compounds and xenobiotics of wastewaters using aerobic granular biomass	According to the present invention, it can provide a kind of when in order to handle the method that contains ammonia-state nitrogen waste water and carry out promotion when biological nitrogen is removed nitration reaction.	[159]
Purification of soil contamination using bacterial strain	EP 2 788 512 B1	Warszawa-Poland; European Patent	Removal of contaminants from soil, as well as a method of soil treatment	The present solution is a natural method of removing hazardous pollutants from the environment without introducing synthetic products.	[149]
Soil and Plant remediation using Atrazine degrading bacteria	CN104762227A	China; Chinese Patent	atrazine degradation-	The bacterium *Arthrobacter ureafaciens liulou* 1 (CGMCC 9667) possesses a unique combination of high atrazine-degrading activity and can colonize plant roots after seed inoculation and traits of plant growth-promoting bacterium.	[160]
Xenobiotic metabolism and associated enzyme	US 2019/0100792 A1	United States; United States Patent	Probes for specifically identifying target active enzymes involved in xenobiotic metabolism	The activity-based probes labeled only their target active enzymes involved in xenobiotic metabolism and therefore provide a measurement of true protein functional activity rather than transcript or protein abundance.	[150]
Bioremediation of xenobiotics in the honey bee hive	US2021378263A1	United States; United States Patent	GE bacteria can hydrolyze ester bonds or remove a carboxyl group	Described herein are engineered cells, enzymes, methods of use, and bee bread incorporating engineered cells and enzymes as described herein to address honey bee hive contamination	[161]
In-vitro model of the human gut microbiome to understand the Impact of xenobiotics	US20200370005	United States Patent	Modifications of xenobiotics by intrinsic gut microbiota	The model facilitates metabolic modeling and enables a better understanding of the structure and function of the human gut microbiome and modifications of xenobiotics by intrinsic gut microbiota, such as biotransformation and bioaccumulation.	[162]

## 6. Conclusions and Future Perspective

Omics approaches are an effective way to understand environmental toxicology and its remediation by employing a hybrid or integrated approach to decipher various effects of xenobiotics and other pollutants on flora, fauna including various ecosystems. The advantages include a better understanding of catabolic genes, degradative enzymes and involved metabolic pathways. In xenobiotic-contaminated soil/water ecosystems, microbial communities have the potential to play an influential role in mediating the successful biodegradation processes. Various molecular techniques provide potential measures to tackle the in-depth assessment of microbial communities at all levels, from the gene to molecule and organism to ecosystem. Many microbes with strong catabolic capability have been identified and described. The omics technique has uncovered many enzymes, especially those produced by unculturable microbes. These innovative steps have discovered various biocatalysts that are organically fitted to industrial restrictions. In this review, several patents have been discussed that employed either single isolates or mixed microbial strains to biotransform xenobiotics from contaminated environments. Resistant microbial technologies must be considered from a practical perspective; however, there is still some controversy on their field applications.

However, more research is required to accomplish exceptional advancements in bioremediation by developing novel genetically modified strains with potent catabolizing genes to have xenobiotics-free ecosystems. Furthermore, the combined approach of green nanotechnology and microbe-mediated bioremediation must be given close attention to combat xenobiotic pollution. Sustainable policies should be developed frequently using contemporary technologies; they need support from government, policymakers, and stakeholders.

## Figures and Tables

**Table 1:** Major xenobiotic compounds and their effects.

Xenobiotic Compounds.	Possible Effects	Consequences Observed	References
**Polychlorinated biphenyls (PCBs)** PCB-156,180,194	Pediatric neurological disorder	Wide range of neural abnormalities i.e., Abnormal reflexes and neural tissue damage	[21,22]
**Halocarbons** CFCs, H(C)FCs, CH_3_CCl_3,_ CCl_4,_ CFC-12 HFC-134a	Global warming and climate change	Loss of biodiversity and habitat destruction	[23,24]
**Synthetic polymers**	Accumulation of PVC and PP products	Alteration in the food chain and food webs, aquatic and soil pollution	[25,26]
**Pharmaceuticals** Analgesics, Antibiotics, Antiepileptic, Antiseptics, Beta-blocker, estrogenic drugs	Cellular and tissue damage	Adverse effect on the reproductive potential of aquatic, terrestrial and arboreal animals, Lethal effect on scavengers	[27]
**Polycyclic Aromatic hydrocarbons** PAHs	Aquatic and avian ecosystem toxicity	Genotoxicity, oxidative stress, immunosuppression and hormonal disorders	[28]
**Polybromonated biphenyls** PBBs	Adverse effects on hormone T3 and T4 secretion	Disorders of the thyroid gland and related hormones	[29]
**Pesticides** Herbicides, Fungicides and Insecticides	Biomagnification and bioaccumulation hazards	Endocrinal anomalies, embryonic cell toxicity in aquatic animals	[30]
**Heavy metals**	Nephrotoxicity, hepatotoxicity, contamination of water tables, aquatic water	Metabolic disorder, cellular and organ damage and a variety of carcinogenic effects	[31]

**Table 2:** List of catabolic genes identified recently for xenobiotic pollutants through various omics approaches.

Genes Identified	Xenobiotic	Likely Pathways	Source	Approaches	References
alkB, alkM, LadA, GSTs, and pcaG	Polycyclic aromatic hydrocarbon (PAH) degradation and n-alkanes	Alkane monooxygenase catalyzes the terminal oxidation of n-alkanes. Ring-hydroxylating dioxygenase degrade PAH	Contaminated soil	Shotgun metagenomic	[101]
abmG and anta	PAH	abmG encodes 2-aminobenzoate-CoA ligase which converts 2-Aminobenzoate to 2-Amino-benzoyl-CoA. The 2-Amino-benzoyl-CoA is transformed into Benzoyl-CoA, Anthranilate 1, 2-dioxygenase encoded by antA gene converts 2-Aminobenzoate to catechol	Polluted river	MinION shotgun sequencing	[102]
nemA, dsrA and dsrB	Nitrotoluene	Trinitrotoluene (TNT) was probably transformed via 2,4,6-TNT	Polluted river	MinION shotgun sequencing	[102]
tceA and vcrA	Trichloro-ethane	Reductive dechlorination of TCE to ethene	Dechlorinated enrichment culture	Transcriptomics	[103]
Nph	4-nitrophenol (4-NP)	Breakdown of 4-NP into acetyl co-A and succinate by nitrocatechol	*Rhodococcus* sp. Strain BUPNP1	Genomic and transcriptomics	[104]
akb, phe and prm	o-xylene	Transformation of o-xylene to 3,4-dimethylphenol and 2-methylbenzylalcohol	*Rhodococcus* *opacus* R7	Genomics	[105]

**Table 3:** Bacterial Enzymes involved in the transformation of various aliphatic and aromatic hydrocarbons.

Xenobiotic	Bacteria	Enzyme	Mechanism of Degradation	Novelties/Inventions	References
**Aliphatic hydrocarbons**	*Xanthobacter* *autotrophicus* GJ10 *Rhodococcuserythropolis* *R. erythropolis* Y2 (England) *R. rhodochrous* NCIMB13064 *Corynebacterium strain* m15				[130]
Haloalkane (1, 2-dichloroethane)		Haloalkane dehalogenase (DhlA)	Nucleophilic substitution reaction to catalyze the displacement of Cl^−^	The genes encoding alkane oxidation in *P. oleovorans* GPo1 are located on the OCT-plasmid in two operons. It indicates the horizontal transfer of catabolic genes across the gram-border. The study emphasizes that horizontal mobilization is faster than the generation of novel catabolic pathways evolved by nature.	
Medium- and long-chain alkanes	*Pseudomonas* *oleovorans* GPo1	Alkane hydroxylase (AlkB, AlkM)	Oxidation of the terminal carbon atom yielding an alcohol		
Sterol	*R. jostii* RHA1	Oxygenase	Catalyzes the hydroxylation and possibly further oxidation of the C26 atom of sterols	Protein fusion strategies used to identify novel activities of cytochrome P450 for biotransformation	[131]
**Aromatic hydrocarbons**		Laccase			[132]
Azo dyes	*Ganoderma* sp.		Oxidize phenolic and methoxyphenolic acids, decarboxylate them and attack their methoxy groups	PCR and cloning approach using basidiomycetes specific primers determine the diversity of laccase and peroxidase-encoding genes, revealing the occurrence of several laccase isozymes.	
Estrogen	*Pseudomonas putida* strains		Ability to remove organic substrate electrons and ultimately reduce dioxygen molecules	This study recommends the use of the consortium of versatile laccase and peroxidase-based biocatalyst for complete removal of multiple estrogens at faster rates.	[133,134]
**Nitro aromatic** **Compounds**					[79,135]
(2-nitrophenol, 4-nitrobenzoic acid, 2-nitro-benzaldehyde, and 3-nitrophenol)	*Xenophilus azovorans* KF46F *Enterococcus faecalis* *Geobacillus stearothermophilus* *Pseudomonas* KF46	Azoreductases	Reduction of azo-bonds	The metaproteomics approach was employed to find out the microbial key players in compost-treated bioremediation	
Catechol and chlorocatechol	*Pseudomonas* sp.	Chlorocatechol 2,3-dioxygenase	Catechol is first transformed into a ring-cleaved product, i.e., 2-hydroxymuconic semialdehyde.		
Protocatechuate	*Acinetobacter calcoaceticus* *Nocardia* sp. *Buttiauxella* sp. S19-1	Protocatechuate 3,4 Dioxygenase	Cleave between the two hydroxyl substituents of protocatechuic acid; with the incorporation of molecular oxygen to form β-carboxymuconate	The study identifies the upregulation of BuP34O (a gene encoding for protocatechuate 3,4-dioxygenase—P34O, a key enzyme in the β-ketoadipate pathway) during TNT degradation.	
**Polyaromatic** **hydrocarbons**	*Pseudomonas putida* (strains: NCIB 9816-4, G7, AK-5, PMD-1, and CSV86), *Pseudomonas stutzeri* AN10, *Pseudomonas fluorescens* PC20, *and other spp.* (ND6 and AS1)				[136]
Naphthalene		Naphthalene dioxygenase (NDO) and ring-hydroxylating dioxygenase	Oxidation of one of the aromatic rings of naphthalene using molecular oxygen	The study presents insights into strain optimization for competent, rapid, and complete bioremediation. The study also highlights that understanding at the biochemical and molecular levels will help identify a suitable host that can be further genetically engineered for efficient bioremediation of priority pollutants	

**Table 4:** Fungi and their working enzymes involved in Xenobiotic transformation.

Xenobiotic	Fungi	Enzyme	Mechanism of Degradation	Novelties/Inventions	Reference
**Aromatic Hydrocarbon** β-lactam	*Fusarium verticillioides*	Lactamases	It hydrolyzes an aromatic polyketide into endocrocin-9-anthron	β-lactamase producing genes were widespread, creating a vast reservoir for genetic transfer between soil microorganisms.	[100]
Atrazine	*Bjerkandera adusta*	Laccases, tyrosimases, manganese peroxidases (MnP), manganese independent peroxidases (MiP) and lignin peroxidases	De-alkylation of atrazine results in fragments of adelhyde and ketone	*Bjerkandera adusta* possess high potential with a removal efficiency of the xenobiotic compound (atrazine) up to 92%.	[137]
Atrazine Monocrotophos DDT	*Fusarium* spp.	N-acetyltransferae and N-malonyltransferase	It helps in the detoxification and degradation of aromatic amines	Acetyl coenzyme A- and malonyl coenzyme A-dependent detoxification	[138]
Aromatic compounds, aliphatic hydrocarbons and PAHs	*Trichoderma harzianum*, *Aspergillus fumigatus*, *Cunninghamella elegans*, *Aspergillus niger*, *Penicillium* sp., *Cunninghamella elegans*, *Aspergillus ochraceus*, *Trametes versicolor*, *Penicillium* sp. RMA1 and RMA2 and *Aspergillus* sp. RFC-1	Lactase, LiP, MnP, epoxide hydrolases cytochrome P450 monoxygenase, dioxygenases, protease and lipase	By peripheral degradation pathways organic pollutants are gradually transformed, and many intermediate products are formed	PHA’s molecular structure was altered by the action of the enzyme, leading to the ring-cleavage processes that produced several intermediate components	[139]
Chlorpyrifos	*Cladosporium cladosporioides*	Chlorpyrifos hydrolase, Pectin methylesterase (PME) and polygalacturonase (PG)	Responsible for pectin degradation by catalyzingthe demethoxylation of the homogalacturonan chain of pectin to release methanol and acidic pectin	Studies that have been conducted on C. cladosporioides discovered bioactive compounds including p-methylbenzoic acid, EP and calphostin C as well as enzymes such as PME, PG and chlorpyrifos hydrolase	[140]
Lignin, Polychlorinated biphenyls (PCBs), Petroleum hydrocarbons, PAHs, trinitroluenes, industrial dye effluents, herbicides and pesticides	*Trametes versicolor*, *Phanerochaete chrysosporium*, *Rigidoporous lignosus* and *Pleurotus ostreatus*	Lignin peroxidase, versatile peroxidase, laccase and manganese peroxidise	Helps in the formation of semi-quinone intermediate during the oxidation of lignin-derived hyroquinone by laccase. It cleaves C-C bonds and oxidizes benzyl alcohols to aldehydes or ketones	The non-specific nature of these enzymes makes them capable of degraders a diverse group of environmental pollutants, including dioxins, polychlorinated biphenyls (PCBs), petroleum hydrocarbons, PAHs, trinitroluenes, industrial dye effluents, herbicides and pesticides	[125,141]
Nitroaromatic compounds	*Phanerochaete chrysosporium*	Peroxidases	Degrades various nitroaromatic compounds by initial reduction of the nitro group tohydroxylamines	Bio-transformation of nitroaromatic compounds and their conversion into nontoxic metabolites via their metabolism	[142]
Navy blue HER, Indigoid, triarylmethane, azo-dibenzothiophene, N-ethylcarbozole and carbozole	*Trichosporon beigelii* NCIM-3326, *P. chrysosporium* URM6181 and *Curvularia lunata* URM6179 *Trametes hirsute* and *Coriolopsis gallica*	Laccase	It attacks phenolic subunit and degrades dyes, leading to C𝛼 oxidation, C𝛼-C𝛽 cleavage and aryl-alkyl cleavage	Lowering the amount of dye in the effluent, showing superior rates of decolorization up to 98% and biodegradation rate 96%, respectively	[143]
PAH and PhC	*Aspergillus sydowii* and *Aspergillus destruens*	Laccase and Peroxidase	Degradation of benzo-α-pyrene phenanthrene	This study revealed that in saline synthetic medium, both fungi used benzo-α-pyrene and phenanthrene as sole carbon sources and removed over 90% of both PAH	[144,145]
